# Population and colony structure of an ant with territorial males, *Cardiocondyla venustula*

**DOI:** 10.1186/s12862-019-1448-6

**Published:** 2019-06-06

**Authors:** Susanne Jacobs, Jürgen Heinze

**Affiliations:** Zoology / Evolutionary Biology, Universität Regensburg, 93040 Regensburg, Germany

**Keywords:** Social insects, Colony structure, Population viscosity, Sib-mating, Life history evolution

## Abstract

**Background:**

Many species of social insects have large-scale mating and dispersal flights and their populations are therefore often relatively homogenous. In contrast, dispersal on the wing appears to be uncommon in most species of the ant genus *Cardiocondyla*, because its males are wingless and the winged queens mate in their natal nests before dispersing on foot. Here we examine the population structure of *C. venustula* from South Africa. This species is of particular interest for the analysis of life history evolution in *Cardiocondyla*, as it occupies a phylogenetic position between tropical species with multi-queen (polygynous) colonies and fighting males and a Palearctic clade with single-queen colonies and mutually peaceful males. Males of *C. venustula* exhibit an intermediate strategy between lethal fighting and complete tolerance – they mostly engage in non-lethal fights and defend small territories inside their natal nests. We investigated how this reproductive behavior influences colony and population structure by analyzing samples on two geographic scales in South Africa: a small 40 × 40m^2^ plot and a larger area with distances up to 5 km between sampling sites in Rietvlei Nature Reserve near Pretoria.

**Results:**

Colonies were found to be facultatively polygynous and queens appear to mate only with a single male. The extraordinarily high inbreeding coefficient suggests regular sib-mating. Budding by workers and young queens is the predominant mode of colony-founding and leads to high population viscosity. In addition, some queens appear to found colonies independently or through adoption into foreign nests.

**Conclusion:**

While *C. venustula* resembles tropical *Cardiocondyla* in queen number and mating frequency, it differs by the absence of winged disperser males. Dispersal by solitary, mated queens on foot or by short flights and their adoption by alien colonies might promote gene flow between colonies and counteract prolonged inbreeding. The abundance of suitable habitat and the high density of nests facilitate the spread of this species by budding and together with the apparent resistance against inbreeding make it a highly successful pioneer species and invader of degraded and man-made habitats.

**Electronic supplementary material:**

The online version of this article (10.1186/s12862-019-1448-6) contains supplementary material, which is available to authorized users.

## Background

The population structure of social insects is strongly shaped by their specific life-histories, in particular their mating behavior, dispersal abilities, and mode of colony founding. Many ant species are characterized by large-scale mating and dispersal flights and lack strong spatial population structure (e.g., [1–3]). In others, young queens mate in or near their natal nests and establish new colonies close by. This leads to high differentiation among populations at least at the level of maternally inherited mtDNA (e.g., [4–7]). Gene flow and outbreeding in the latter case are typically promoted by the dispersal of winged males [8, 9].

Winged males have been replaced by wingless “ergatoid” males in most species of the ant genus *Cardiocondyla* [10–13]. Mating occurs inside the nest and mostly involves sibs, and the frequent occurrence of solitarily walking winged and dealate queens (which have lost their wings after mating) [13] suggests limited dispersal capability and a highly viscous population structure. This stands in striking contrast to reports that *Cardiocondyla* are often among the first ants colonizing disturbed or rehabilitated habitats and that several species are highly successful “tramp species” with an almost worldwide distribution [14–17]. While the reproductive behavior of *Cardiocondyla* has been studied in detail in a number of species (e.g., [18–21], little is known about the genetic structure of populations and how far queens disperse.

The African species *Cardiocondyla venustula* is of particular interest in this context because of its placement in the phylogeny of the genus: it is positioned between tropical taxa, where colonies typically contain multiple, singly-mated queens and wingless males engage in fatal fighting over access to freshly emerging virgin queens, and a Palearctic clade where colonies have a single, multiply-mated queen per colony and mutually tolerant males [12, 13]. Analyzing the social and genetic structure of *C. venustula* colonies and populations therefore is of considerable importance for unravelling the evolution of different life histories in this genus.

Behavioral studies revealed that males of *C. venustula* are always ergatoid and show an “intermediate” behavior between fatal fighting and complete tolerance: they defend small areas in the nest against nestmate males, engaging in regular, but typically non-lethal fighting with nestmate males [22, 23]. Female sexuals (young, winged queens) were observed interacting with several males, but it remained unclear if they mated multiply. However, from male territoriality we hypothesized that queen mating frequencies would be low as in species with lethally fighting males.

Field studies gave conflicting results concerning queen number: while Wheeler [24] reported on regular single-queening in an introduced population in Puerto Rico, our own observations revealed the regular presence of multiple queens per colony both in introduced and native populations [23, 25]. The finding of dense populations of *C. venustula* near Pretoria and Bergville, South Africa, allowed us to conduct detailed analyses of the genetic structure of colonies and populations and how they are influenced by male territoriality and female dispersal on foot.

## Methods

### Sampling and field observations

Samples for DNA analysis were collected in Rietvlei Nature Reserve, Pretoria, Gauteng, South Africa, and Hlalanathi Drakensberg Resort, Bergville, KwaZulu-Natal, South Africa, in February and March 2013. Workers were easily spotted when foraging on bare or sparsely vegetated ground, e.g., on unpaved parking lots. They were followed back to their nests, which consisted of two or three pea-sized holes in the ground down to a depth of 20 cm [25]. Subsequently, these nests were carefully excavated and the ants were either directly transferred into a tube with 100% ethanol or transferred to the laboratory alive for behavioral experiments. Of the latter colonies, a few individuals were also stored in 100% ethanol immediately after collection.

To analyze genetic population structure, relatedness and dispersal on a small scale, we measured the distances between all 57 colonies found in February 2013 in an area of 40 × 40 m^2^ at the main gate of Rietvlei Nature Reserve (Riet: S 25.88250, E 28.26417, 1515 m). A few colonies from sampling areas in and near Rietvlei Nature Reserve, 0.5 to 5.5 km away from the plot at the entrance building, were used to estimate gene flow and population structure on a larger scale (C: Rietvlei Coffee Shop, *n* = 13, S 25.87722, E 28.30083, 1511 m; CC: Coots Corner Bird Hide parking lot, *n* = 4, S 25.88139, E 28.26861, 1477 m; IV: Island View Bird Hide parking lot, *n* = 8, S 25.87639, E 28.28056, 1481 m; MD: Marais Dam parking lot, *n* = 8, S 25.90528, E 28.30833, 1511 m; PH: Pheasant Hill B&B, *n* = 2, S 25.88722, E 28.25778, 1508 m). Additionally, colonies from Hlalanathi Drakensberg Resort were used for estimation of relatedness and inbreeding (*n* = 82, S 28.65903, E 29.03136, 1288 m).

### DNA extraction, microsatellite genotyping, and mt DNA sequencing

Before DNA extraction, samples were frozen in liquid nitrogen and crushed with a pestle in order to destroy the hard cuticle. Thereafter, DNA was extracted from older or damaged whole ants or pupae using a CTAB protocol [26] and from fresh and intact specimens using the Macherey Nagel XS Tissue Kit following the protocol for tissue samples.

Seven microsatellite markers developed for *Cardiocondyla* were found to be sufficiently polymorphic for population genetic analysis in *C. venustula* (CE2-3A, CE2-4E, CE2-5D, CE2-12D [27]; Card 8 [28]; Cobs 3 and Cobs 13 [29], see Table 1 for details). PCRs were conducted with a total volume of 15 μl using 7.5 μl GoTaq PCR mix (Promega, Madison, WI), 5 μl water, 0.5 pm/μl of each primer, and 1 μl of the DNA solution. Forward primers were end-labelled with fluorescent dye (FAM, Eurofin Genomics, Ebersberg). PCRs were run using an initial denaturation step at 94 °C for 4 min followed by 36 cycles with 70 s at 94 °C for denaturation, 45 s at the specific annealing temperature, and 25 s at 72 °C for elongation, and a final elongation step at 72 °C for 3 min. Fragment lengths were determined on an ABI PRISM using a TAMRA labelled size standard. Alleles were scored using Genescan 3.1 software (PR Biosystems).

**Table 1:** Number of alleles N_A_, allele sizes, expected (H_exp_) and observed heterozygosity (H_obs_), and inbreeding coefficient for microsatellite markers in the ant *Cardiocondyla venustula* from two populations in South Africa

Marker	N_A_	Allele size	H_exp_	H_obs_	F
Hlalanathi					
Ce2-3a	10	80–102	0.723	0.198	0.728
Ce2-4e	2	101–107	0.426	0.051	0.882
Ce2-5d	4	192–198	0.283	0.171	0.397
Ce2-12d	15	132–206	0.847	0.207	0.756
Card8	6	121–133	0.702	0.171	0.758
Cobs3	11	103–139	0.664	0.207	0.689
Cobs13	7	72–88	0.562	0.049	0.914
Rietvlei					
Ce2-3a	4	84–92	0.043	0.043	−0.010
Ce2-4e	1	107	–	–	
Ce2-5d	1	194	–	–	
Ce2-12d	2	132–134	0.366	0.100	0.728
Card8	2	121–127	0.291	0.132	0.549
Cobs3	9	121–139	0.691	0.253	0.636
Cobs13	4	76–84	0.488	0.228	0.533

We analyzed a 795 bp-fragment of the COI/COII gene. Amplification of mtDNA was conducted in a total volume of 25 μl using 12.5 μl GoTaq PCR mix, 8.5 μl water, 0.5 pm/μl of each primer, and 1.5 μl of the DNA solution. Due to amplification problems with standard primers, we designed new primers using conserved regions in the previously obtained COI/COII-sequences: COICv-f (5′-ATTATCGCCGTCCCTACAGG-3′) and COICv-r (5′-TCGGATGGGGAAGTTATAAGGT-3′). PCRs were run using an initial denaturation step at 94 °C for 4 min followed by 39 cycles with 75 s at 94 °C for denaturation, 45 s at the specific annealing temperature, and 90 s at 72 °C for elongation, and a final elongation step at 72 °C for 7 min. PCR products were purified using the Macherey-Nagel Gel and PCR clean-up kit following the manufacturer’s instructions and sent to LGC Genomics, Berlin, for sequencing. The trace files of obtained sequences were checked by eye for sequence quality.

### Analysis of mating frequency

Field observations and dissections suggested that colonies of *C. venustula* might contain multiple, fertile queens. Polygyny was additionally corroborated by the analysis of workers by microsatellite genotyping. In addition, we investigated by COI sequencing whether colonies may contain individuals belonging to several unrelated lineages.

Queens and their worker offspring from natural colonies were genotyped to determine queen mating frequencies. However, these genotypes did not always allow determining whether workers were offspring of one queen, which had mated with several related males, or offspring of multiple related queens sharing the same mitochondrial haplotype, of which several might have been missed during or had died before collection. We therefore investigated whether female sexuals mate with multiple males in the laboratory. We set up experimental colonies, each consisting of ten workers, brood, and a queen pupa, in small nests (diameter appr. 1.5 cm) and later added one male each from two unrelated colonies with different genotypes, using colonies from both the populations at Rietvlei and at Hlalanathi, KwaZulu-Natal. To avoid males killing each other we first kept male pupae separately in nests with some brood and workers from their natal colony. After they had eclosed and hardened their cuticula, they were transferred into the experimental colonies with the young queen pupae. Experimental colonies, in which one of the males had died prematurely, were excluded from the analysis. Thus, each female sexual was provided with simultaneous access to two males until it was mated. Mating events are inconspicuous and difficult to observe [23]. We therefore waited for several days until all queens had shed their wings – which they usually do only after mating - and then stored them in 100% before genotype analysis of stored sperm following Lenoir et al. [30].

As the small amount of sperm DNA might not always give a reliable estimate of mating frequencies (because of large allele dropout or non-detection of alleles due to varying amounts of sperm from individual males), we also determined mating frequency from offspring genotypes. To do so we transferred ten additional, dealate queens into small colonies containing 20 workers and large larvae or worker pupae. From six of these queens we managed to collect and genotype five to eight 2nd instar or larger larvae. Thereafter, the queens were killed and genotyped.

### Population genetic analyses

We used the software STRUCTURE [31] to find potential population structure on two scales, the 40 × 40m^2^ plot and the whole Rietvlei Reservation Area. The results were analyzed with STRUCTURE harvester [32] in order to find the optimal number of clusters (K). In addition, we assessed K with the function find.clusters implemented in the package “adegenet” [33] in R [34]. Population genetic parameters, intra- and intercolonial relatedness were calculated in SpaGeDi 1.5a [35]. Isolation by distance was tested via Mantel test comparing genetic and geographic distance matrices with the software Arlequin 3.1 [36] with 10,000 permutations. As a measure for genetic distance in the 40 × 40m^2^ plot codominant pairwise distances were calculated in GenAlEx 6.5 [37], for the inter-population test, Slatkin’s linearized F_ST_ [38] was calculated as implemented in Arlequin. F-statistics were calculated using GDA 1.1 with bootstrapping over loci to obtain confidence intervals [39]. Significance of pairwise differentiation between sampling sites was calculated by permutation in Arlequin [36]. Microsatellites were tested for the existence and frequencies of null-alleles using INEST 2.2 [40].

## Results

### Field observations

Solitary queens, both winged and dealate, were regularly seen walking on the ground; one winged queen was observed flying for approximately 20 cm from the top of a blade of grass. These observations and dissections of winged, dispersing queens in the lab suggest short-range dispersal by queens both before and after mating (see also [25]).

Of the 92 colonies collected in Rietvlei Nature Reserve, 30 contained a single dealate queen, 17 contained multiple, up to seven dealate queens, and the rest were queenless. The latter were probably incompletely sampled and were directly transferred into EtOH for genetic analyses.

### Population structure

We obtained microsatellite genotypes of one worker each for 92 colonies from Rietvlei Nature Reserve (57 colonies from main gate, 13 colonies from C, four colonies from CC, eight colonies from IV, eight colonies from MD, two colonies from PH). Estimation of null allele frequencies in INEST did not support the existence of null alleles in any of the markers.

**Fig. 1:**
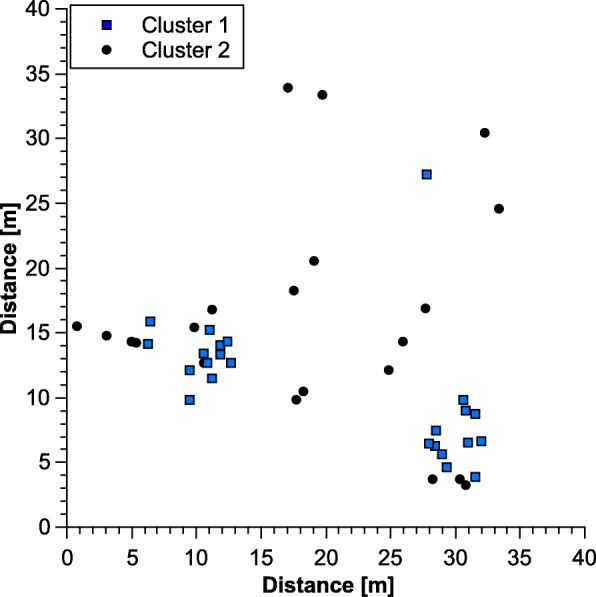
Distribution of clusters (K = 2) found by STRUCTURE among nests of the ant *Cardiocondyla venustula* in the 40 × 40 m^2^ grid at the main gate of Rietvlei nature reserve, South Africa. Coordinate points (circles and triangles) represent one sampled colony each

Mean F_ST_-values indicated considerable differentiation between collecting sites (mean F_ST_ = 0.308, 95% confidence interval 0.136–0.456; Table 2). F_ST_-values were positive for all pairs of sites, and permutation tests revealed significant differences in 11 of 15 pairwise comparisons. STRUCTURE analysis proposed two clusters in the intensively sampled 40 × 40 m^2^ plot. While the “find.clusters” algorithm in DAPC did not provide clear clustering, manually setting the number of clusters to two revealed the same grouping as the one calculated by STRUCTURE. Plotting genetic clusters against sampling location in the 40 × 40 m^2^ plot showed that cluster 1 (blue squares in Fig. 1) appeared to be mostly concentrated in two distinct spots in the sampling area while cluster 2 (black circles in Fig. 1) was distributed relatively evenly over the whole sampling area.

**Table 2:** Pairwise F_ST_-values and geographic distance between sampling sites of the ant *Cardiocondyla venustula* at Rietvlei Nature Reserve, South Africa.

	Main gate *n* = 57	PH *n* = 2	MD *n* = 8	C *n* = 12	IV *n* = 8	CC *n* = 4
Main gate	–	0.8 km	5.1 km	3.7 km	1.8 km	0.5 km
PH	0.15	–	5.5 km	4.5 km	2.6 km	1.2 km
MD	0.21^a^	0.56^a^	–	3.2 km	2.0 km	4.8 km
C	0.01	0.15	0.24^a^	–	2.1 km	3.3 km
IV	0.49^a^	0.78^a^	0.86^a^	0.53^a^	0	1.3 km
CC	0.42^a^	0.54	0.62^a^	0.39^a^	0.69^a^	–

Significant F_ST_ -values marked with ^a^, significance level 0.05

**Fig. 2:**
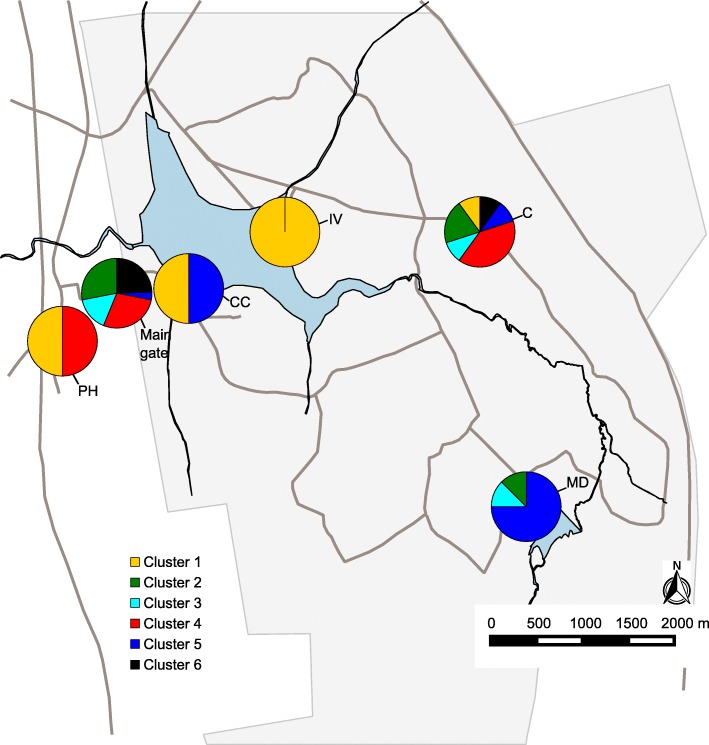
Location of sample sites with distribution of clusters (K = 6) found by STRUCTURE among nests of the ant *Cardiocondyla venustula* at Rietvlei nature reserve, South Africa. Pie charts represent sample sites (not to scale) with the relative abundance of samples assigned to the respective cluster. Rietvlei nature reserve is depicted in light gray, water surfaces are depicted in darker grey. Sample site abbreviations: PH = Pheasant Hill B&B, CC = Coots Corner Bird Hide parking lot, IV = Island View Bird Hide parking lot, C = Rietvlei Coffee Shop, MD = Marais Dam parking lot

Across all collecting sites in Rietvlei, STRUCTURE suggested six clusters. Similar to the analysis in the smaller plot, “find.clusters” in DAPC did not reveal clear clustering. However, when K was set to six, results were concordant with those from STRUCTURE. While F_ST_-values suggested significant differentiation among sampling sites, individuals from different sampling sites clustered together in structure analysis (Fig. 2). A comparison of pairwise genetic and geographic distances suggested a weak trend of isolation by distance in the 40 × 40 m^2^ plot (*p* = 0.06, *r*^*2*^ = 0.01). When comparing pairwise F_ST_-values and geographic distances within the whole sampling area, no such effect was visible (*p* = 0.40, *r*^*2*^ = 0.01).

**Fig. 3:**
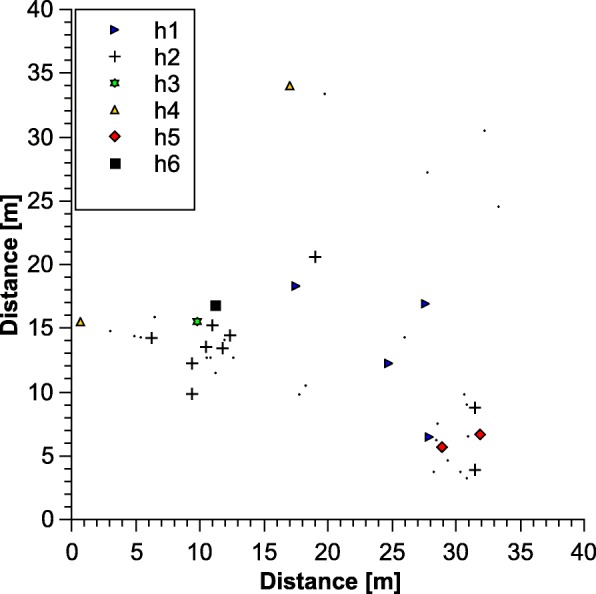
Distribution of mtDNA-haplotypes of the ant *Cardiocondyla venustula* in the 40 x 40 m^2^ grid at main gate of Rietvlei nature reserve, South Africa. Coordinate points represent one sampled colony each, dots represent samples used in microsatellite, but not in mtDNA analysis

**Fig. 4:**
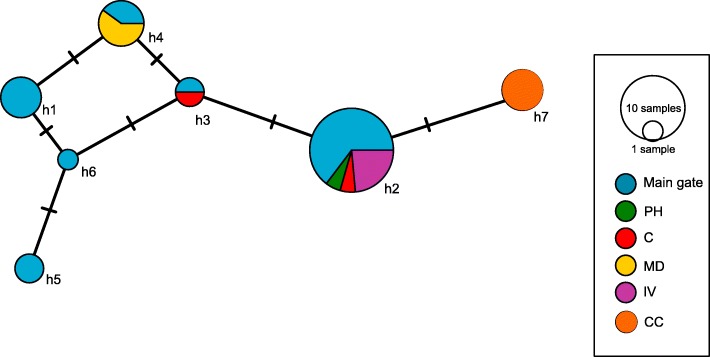
Haplotype network for the 795-bp fragment of the COI-gene of the ant *Cardiocondyla venustula* at Rietvlei nature reserve, South Africa. Pie charts represent one haplotype each, sample sites are represented by the respective color/signature. Sample site abbreviations: PH = Pheasant Hill B&B, CC = Coots Corner Bird Hide parking lot, IV = Island View Bird Hide parking lot, C = Rietvlei Coffee Shop, MD = Marais Dam parking lot

Due to amplification problems, mtDNA sequences were available only for a subset of 44 individuals from 35 colonies (21 from main gate, two from C, four from CC, four from IV, three from MD, one from PH). In the 40 × 40m^2^ plot we found six haplotypes, one of which, h2, exhibited a similar distribution to that observed in microsatellite cluster 1, occurring mainly in two spots in the sampling area. The other haplotypes were scattered (Fig. 3). Across all sampling sites, seven haplotypes were found (GenBank Accession numbers MK138574 – MK138580). Interestingly, we found only a single haplotype at several of the less intensively sampled sites (Fig. 4). Comparison of genetic clusters found by STRUCTURE analysis of the microsatellite genotypes with those found in mitochondrial haplotypes revealed an independent distribution of both marker types (Mantel test for correlation between F_ST_-values: *p* = 0.42, *r*^*2*^ = 0.005).

Isolation by distance was similarly low in mtDNA markers as in microsatellite markers. For the 40 × 40 m^2^ plot, isolation by distance was significant but weak (*p* = 0.05, *r*^*2*^ = 0.02). Between the sampling sites, no significant effect was found (*p* = 0.40, *r*^*2*^ = 0.005). Maternally inherited mtDNA and the bi-parentally inherited genomic microsatellites and their spatial distribution thus revealed only subtle differences, suggesting a female-biased dispersal and supporting the observations of dispersing queens in the field.

### Relatedness and colony structure

For the analysis of relatedness and colony structure, we obtained microsatellite genotypes from 8 to 23 workers per colony, analyzing ten colonies from Rietvlei (117 genotypes in total) and twelve colonies from Hlalanathi (112 genotypes in total). F_IS_ and background allele frequencies were calculated using one individual per colony for all sampled colonies in the respective populations (91 colonies from Rietvlei, 82 colonies from Hlalanathi). Microsatellite genotypes showed high levels of inbreeding in all sampled areas in Rietvlei (overall F_IS_ = 0.608, 95% confidence interval 0.366–0.562) as well as in Hlalanathi (overall F_IS_ = 0.551, 95% confidence interval 0.422–0.614), suggesting 86.1 and 83.1% sib-mating, respectively (following [41]). Though field observations revealed the frequent presence of multiple queens within a single colony (see above), relatedness was usually high (Rietvlei: mean 0.867 ± S.E. 0.079, ranging from 0.030 ± 0.291 to 1 ± 0; 5 colonies only from Rietvlei main gate: mean 0.767 ± S.E. 0.133, ranging from 0.567 ± 0.175 to 0.983 ± 0.018; Hlalanathi: mean 0.786 ± S.E. 0.096, ranging from 0.456 ± 0.185 to 1 ± 0). This matches the high inbreeding coefficient and the assumption that workers may occasionally be offspring of multiple, usually related queens, most of which have mated with the same or several closely related males. Correction for inbreeding [42] yielded a mean relatedness of 0.454 (Rietvlei) and 0.261 (Hlalanathi). The co-occurrence of two different mtDNA haplotypes in workers of a previously studied colony [25] indicates that colonies may contain multiple unrelated queens or occasionally adopt alien workers.

The non-detection error arising from the low variability of microsatellites typical for most *Cardiocondyla*, the high inbreeding coefficient, and the fluidity of colony structure all make it difficult to exactly determine the number of matrilines and patrilines. We therefore staged mating experiments in the lab to determine whether female sexuals (young, winged queens) mate with multiple or single males. In the spermathecae of queens that had been given the possibility to mate with two genetically different males, we did never find more than one paternal allele, i.e., there was no evidence of multiple inseminations. Similarly, the five to eight offspring larvae produced each by the six queens that had been exposed to two males were all offspring of only one father (total number 36 larvae).

## Discussion

The reproductive biology of ants of the genus *Cardiocondyla* with stationary wingless males, mating in the nest, and dispersal on foot by winged, mated and / or unmated queens is quite unusual for ants and social insects in general [13]. As we here show specifically for *C. venustula* from Rietvlei Nature Reserve in South Africa, this leads to high levels of inbreeding and nestmate relatedness.

Unfortunately, the low genetic diversity, already known from previous studies in other *Cardiocondyla* species (e.g. [29, 43, 44]) together with sib-mating and the short lifespan and rapid turnover of queens [45] makes it difficult to resolve colony and population structure to a high degree of accuracy. Nevertheless, our findings are in agreement with previous assumptions about the dispersal biology of this genus, with local mating, short-range dispersal by queens, colony budding, and occasional long-range dispersal by queens on the wing or mediated by human activities. Population viscosity was surprisingly small both at the level of nuclear microsatellites and mtDNA haplotypes, and no significant structure was seen when colonies from more distant collecting sites were included in the analysis.

A detailed survey in a 40 × 40 m^2^ plot with almost 60 colonies showed that colonies with similar microsatellite genotypes and mtDNA haplotypes may occasionally cluster. This matches our field observations: while wingless males are only very rarely observed outside the nest (e.g., [30, 46]), winged and wingless queens are regularly seen walking over distances of a few meters. Winged queens may even be drifted over longer distances by wind [13], explaining the relatively weak isolation by distance. The clumped distribution of cluster 1 and haplotype 2 in the 40 × 40 m^2^ plot suggest that new colonies may be founded by budding, i.e., young queens disperse together with several workers from their natal nest after mating. In the laboratory, new colonies are easily produced by splitting multi-queen colonies (e.g., [19]), supporting the hypothesis that budding is a regular mode of founding in this genus. Whether colonies of *C. venustula* may inhabit several neighboring nests (polydomy), as suggested for populations of this species introduced to Puerto Rico [24, 47], remains unclear. Workers in Rietvlei exhibited aggression or backed off when confronted with a worker from an adjacent nest in a small confined space in the field (S. Jacobs, unpublished observations), making it unlikely that buds remain connected to the natal nest.

The rare co-occurrence of different mtDNA-haplotypes within the same colony [25] might suggest that in addition to colony founding by budding solitarily dispersing queens occasionally may be adopted into alien nests. This would explain the lack of a strong correlation between mtDNA and microsatellite clusters and also promote gene flow between colonies. In the laboratory, groups of multiple queens of *C. venustula* and other *Cardiocondyla* were able to rear larvae ([48], S.J., unpubl. observations). Hence, cooperative founding by unrelated dispersing queens provides an alternative way of colony founding and might also contribute to the existence of multiple mtDNA haplotypes in one colony.

Though F_ST_-values were significant, we could not reveal a clear genetic structure across all sampling sites in Rietvlei Nature Reserves. The population from Island View (IV) did not show any variability in the analyzed markers despite a sufficiently large sample size at least for microsatellite genotyping. This might indicate that populations are occasionally founded by only one or a few related queens (with or without workers). Isolated patches might be colonized via several stepping stones of small, sparsely vegetated patches. In addition, the worldwide spread of several *Cardiocondyla* tramp species [13, 49–51], including *C. venustula*, suggests that the accidental transfer of solitary queens or colony fragments with soil, seedlings, or garbage may ease the colonization of new habitat and may have contributed to these apparent founder effects. The network of unpaved roads in Rietvlei Nature Reserve may also have facilitated the dispersal of *C. venustula* as such roads provide the ideal habitat for these ants.

The inbreeding and relatedness coefficients obtained by microsatellite genotyping for nestmates of *C. venustula* were higher than in other *Cardiocondyla* species [28–30, 44], including the related *C. shuckardi* from Madagascar [43]. The inbreeding coefficient suggests more than 80% sib-mating. Generally, *Cardiocondyla* show a high tolerance to inbreeding. In *C. obscurior*, no diploid males were found even after several generations of sib-mating in the lab [52]. In contrast to honeybees and presumably also other social Hymenoptera [53], *Cardiocondyla* does not exhibit single-locus complementary sex determination and inbreeding therefore does not lead to the production of non-viable or sterile diploid males. Resistance towards inbreeding and genetic impoverishment has previously been suggested to facilitate the successful establishment of small propagules in novel environments [16, 54–57], and our study again highlights that sib-mating in *Cardiocondyla* is not a consequence but a precondition of invasiveness (see also [58]).

Nevertheless, exclusive inbreeding may occasionally have adverse effects also in *Cardiocondyla*, including reduced queen life span and a lower fecundity of males [52]. These are presumably lessened by occasional outbreeding events, e.g., through the adoption of alien queens as described above. Winged queens have regularly been observed to disperse on foot, and some of them may enter and mate in alien nests, similar to what has been observed in Palearctic *Cardiocondyla* [30, 44]. Wingless males of *C. venustula* may therefore not only compete for mating with their sisters [22, 23] but also for mating with alien queens. The presence of multiple fertile queens in field and also lab colonies (unpublished data) suggests that *C. venustula* is facultatively polygynous, in contrast to the obligatorily monogynous Palearctic clade. While workers typically are expected to oppose the adoption of alien queens on relatedness grounds (e.g., [59, 60]), *C. venustula* workers would be closely related to an adopted queen’s offspring in case it has mated with one of the colony’s males.

The low variability of genetic markers and the presumed high relatedness among nestmate males did not allow determining the mating frequency of field-collected queens. Sperm and offspring analyses suggest that queens, which in the lab had been given the chance to mate with two genotypically distinct males, mated only once. Monogamy in *C. venustula* is in accordance with males successfully increasing their fitness by defending small “territories” inside the nests and monopolizing mating with female sexuals inside the nest. While obligatorily monogynous *Cardiocondyla* are polyandrous [28, 30, 44], facultatively polygynous *C. obscurior* [29] and, as we here show, *C. venustula* appear to be monandrous. This matches previous observations of the correlation between queen number and mating frequency in other ants (e.g., [61]).

## Conclusion

Our study shows that *C. venustula* resembles other tropical species of the genus in the presence of multiple queens per nest and single mating by queens. Though lethal fighting among *C. venustula* males appears to be uncommon, the aggressive defense of small areas in the nest appears to allow them to monopolize mating with many female sexuals. The very high levels of inbreeding and nestmate relatedness indicate that most matings involve siblings, but the co-occurrence of multiple mtDNA haplotypes in a single colony suggests that dispersing queens may occasionally be adopted by alien colonies. This can promote gene flow and alleviate the negative effects of prolonged inbreeding. Dispersal by young queens over short distances and budding appear to be the prevalent modes of colony founding. Though it still remains to be clarified how long-range dispersal is achieved, the preference of *C. venustula* for ruderal, degraded patches with sparse vegetation certainly facilitates the colonization of novel habitats, such as rehabilitated mines, parks, or roadsides. Human-assisted transfer in potted plants may have allowed it to spread from its Afrotropical origin to Florida and several Caribbean and Pacific Islands [11, 17] and may also have contributed to the limited isolation by distance across all collecting sites in Rietvlei.

## Additional file

Additional file 1: Raw data of the population genetic analysis of the ant *Cardiocondyla venustula* from South Africa. (XLSX 40 kb)

## Data Availability

mtDNA sequences have been uploaded to GenBank (accession numbers MK138574 – MK138580). The results from microsatellite analyses are available as Additional file 1 Material.
